# The Effect of Polyunsaturated Fatty Acid (PUFA) Supplementation on Clinical Manifestations and Inflammatory Parameters in Individuals with Sjögren’s Syndrome: A Literature Review of Randomized Controlled Clinical Trials

**DOI:** 10.3390/nu16213786

**Published:** 2024-11-04

**Authors:** Catarina Bento da Nave, Paula Pereira, Maria Leonor Silva

**Affiliations:** 1 Egas Moniz School of Health and Science, 2829-511 Caparica, Almada, Portugal; 115138@alunos.egasmoniz.edu.pt; 2 Nutrition Lab, Applied Nutrition Research Group, Egas Moniz Center for Interdisciplinary Research (CiiEM), Egas Moniz School of Health & Science, 2829-511 Caparica, Almada, Portugal; pmpereira@egasmoniz.edu.pt

**Keywords:** Sjögren’s syndrome, nutritional supplementation, polyunsaturated fatty acids, omega-3, omega-6

## Abstract

Background. Sjögren’s syndrome is a chronic autoimmune disease that causes dry mouth and eyes and can lead to non-Hodgkin’s lymphoma in 5–10% of cases after 10 years. Clinical trials have shown that the oral administration of polyunsaturated fatty acids (PUFAs) seems to have a beneficial effect on Sjögren’s syndrome. Aim. This literature review provides an overview of the effects of PUFA supplementation on clinical manifestations and inflammatory parameters in Sjögren’s syndrome. Methodology. We conducted a literature review using the PubMed, Biomed Central, and Cochrane Library electronic databases and using search terms “Sjögren” AND “omega-3”; and “omega-6” AND “fatty acids” AND “oil”. This literature review followed the PRISMA guidelines and included randomized clinical trials in humans with or without a control group using the oral administration of PUFA. Results. From 26 articles found in the databases, a total of 6 articles were included. Of these six trials, five trials showed an effect on clinical manifestations and three trials on inflammatory parameters. Most of the studies did not show a significant effect on the parameters analyzed. One study showed a significant improvement in dry keratoconjunctivitis compared to the control group. The results suggest that PUFAs may improve inflammatory parameters in patients with Sjögren’s syndrome. Conclusions. This literature review supports the idea that the oral administration of PUFA may possess a potential effect on clinical manifestations. However, due to the limited number of studies and the heterogeneity of clinical trial methodology, further investigations should be employed. Understanding the potential mechanism of action of PUFAs on clinical biomarkers in Sjögren’s syndrome may clarify their importance in clinical practice for health professionals.

## 1. Introduction

Sjögren’s syndrome is a type of chronic systemic rheumatoid arthritis caused by lymphocytic infiltration affecting the salivary and lacrimal glands [1]. It is currently the second most common autoimmune disease, followed by rheumatoid arthritis (RA) [2]. The main symptoms include xerostomia, dry mouth or eyes, and the inflammation of the internal organs [2,3]. This disease is usually associated with the production of specific antibodies, such as anti-Ro/SSA and anti-La/SSB [3,4], and can occur independently or in combination with other autoimmune diseases, such as rheumatoid arthritis, septic shock, or autoimmune liver disease [1,2,3]. Primary Sjögren’s syndrome (pSS) can affect any organ system and manifests as extraglandular clinical manifestations (ECMs) [4]. These can be divided into non-visceral and visceral symptoms, caused by neurological, renal, hematological, pulmonary, gastrointestinal, and cardiovascular diseases [5].

Primary Sjögren’s syndrome (pSS) is common in the United States (US) population, with an incidence of 7/100.000 inhabitants [2,6]. The average age of the general population with pSS is approximately 56.16 years old [6], and the female-to-male ratio in the population with pSS is 9:1 [5,7]. Approximately 5–10% of diagnosed patients may develop non-Hodgkin’s lymphoma from B cells (B-type lymphocytes) after 10 years [3]. The prevalence is higher in Europe than in Asia, with no significant difference in gender distribution between continents [5].

Sjögren’s syndrome is characterized by multiple genetic and environmental factors that lead to immunological changes in the immune system, resulting in the development of primary Sjögren’s syndrome [4]. In the adaptive immune system, T lymphocytes produce pro-inflammatory cytokines, while B lymphocytes play a crucial role in the development of lymphoma [1,8,9,10,11]. In the innate immune system, dendritic cells, epithelial cells, and natural killer cells are also important in the pathogenesis of Sjögren’s syndrome, playing an essential role in the production of type I interferons (IFNs), controlling the expression of specific antibodies, modulating cytokine production, and regulating the expression of chemokines [12,13,14,15,16,17].

Currently, there is no single diagnostic test for primary Sjögren’s syndrome (pSS) because of its insidious, slow, and unknown forms, which makes it difficult to identify [3]. To speed up diagnoses, early detection is recommended to reduce the number of damaged salivary and lacrimal glands [3]. Different types of tests are used, including objective, ophthalmological, oral, and subjective tests [3]. Diagnostic criteria include positive anti-SSA/Ro antibodies, salivation, eye color, the Schirmer test, and the salivary flow rate [18].

Beyond the diagnosis, the therapy and prevention of the disease are similarly challenging, where PUFAs may be a viable option for improving clinical manifestations and inflammatory parameters. Therefore, PUFA therapy may promote a better quality of life for people with this syndrome. Published data have shown that polyunsaturated fatty acids (PUFAs) have a potential effect on Sjögren’s syndrome patients [19,20,21]. Efamol is a seed oil from a specially grown strain of the evening primrose that has been used in the treatment of Sjögren’s syndrome. The Efamol supplement is rich in essential components, namely, polyunsaturated fatty acids (omega-6), such as gamma-linolenic acid (GLA), and saturated and monounsaturated fatty acids. Flaxseed oil, due to its unique composition and being a plant-based source rich in omega-3 polyunsaturated fatty acids, whose main constituents are linoleic acid and α-linolenic acid (ALA), is also increasingly valued for its beneficial properties for individual health [22]. It has been found that supplementation with flaxseed oil tends to reduce ocular inflammation [23], just as supplementation with Efamol tends to show significant improvements in dry keratoconjunctivitis and erythrocyte phospholipid levels [21] in individuals with Sjögren’s syndrome.

Although the literature is inconsistent regarding the effects of dietary supplementation, studies have also produced controversial results. The possible beneficial effect of PUFAs is due to their anti-inflammatory and antioxidant properties [24,25]. These compounds are described as having a therapeutic role in alleviating the symptoms of arthritis and other autoimmune diseases [26]. This narrative review provides an overview of the effect of polyunsaturated fatty acid (PUFA) supplementation on clinical manifestations and inflammatory parameters in patients with Sjögren’s syndrome. We also discuss the possible mechanism of action of PUFAs in Sjögren’s syndrome. Our study aims to contribute as a tool for better clinical practice and thus improve the quality of life of Sjögren’s syndrome patients.

## 2. Materials and Methods

### 2.1. Data Search and Eligibility Criteria

Literature searches were conducted from March to July 2024 in the PubMed, Biomed Central, and Cochrane Library databases using the following keywords: “Sjögren” AND “omega-3” AND “omega-6” AND “fatty acids” AND “oil”. No restrictions on publication dates were applied. Preferred Reporting Items for Systematic reviews and Meta-Analyses (PRISMA) guidelines were followed in order to conduct this literature review. Inclusion criteria were formulated prior to the literature search and included randomized clinical trials, humans aged 18 years or older, individuals with a diagnosis of Sjögren’s syndrome, male or female participants, study design including experimental and/or quasi-experimental clinical trials, studies with or without a control group, studies with a dietary intervention based on polyunsaturated fatty acids, and publications in English. Exclusion criteria included non-peer-reviewed articles, proceedings, and letters/comments.

### 2.2. Data Selection and Collection

For the study selection, two independent researchers conducted an abstract review in order to avoid the bias risk, in which the study design, sample, interventions, and outcomes were analyzed. Clinical manifestations and the inflammatory parameters were considered as variables. The main outcomes in this study included fatigue, salivary flow, dry mouth symptoms, mean prostaglandin values, pain, dryness, keratoconjunctivitis, corneal sensitivity, and inflammatory biomarkers. The screening process was based on the review of titles and abstracts, and articles were assessed on the basis of the full text to verify that they met the inclusion criteria. Another independent researcher assessed the studies’ eligibility and clarified in case of disagreement regarding eligibility criteria. The Population, Intervention, Comparison, and Outcome (PICO) framework was included in the main results of this literature review.

## 3. Results

### 3.1. Literature Search and Outcomes

**Figure 1:**
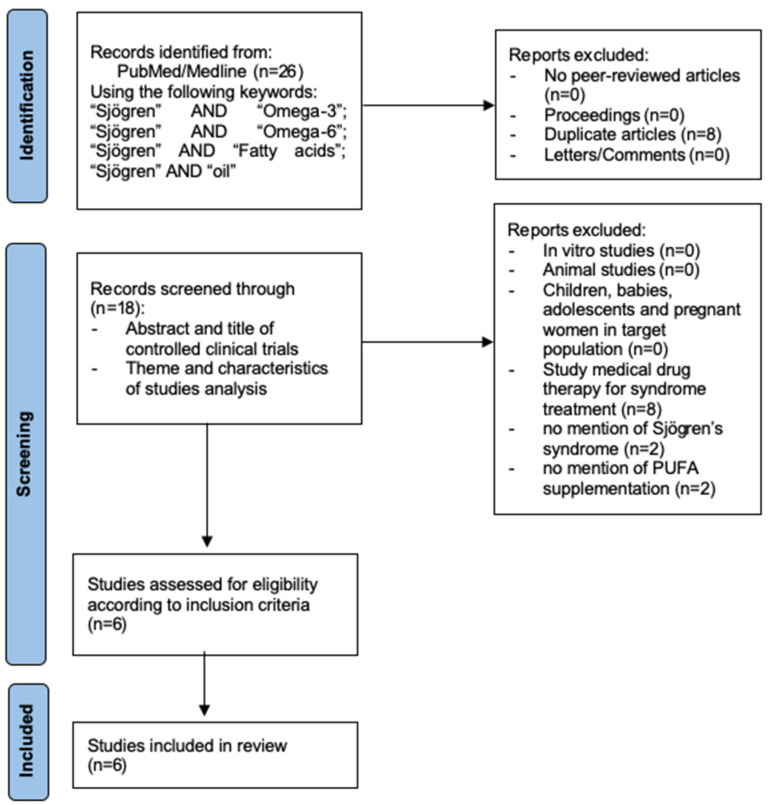
PRISMA flowchart of study selection process.

From the studies found in the databases, a total of 26 publications were identified in this literature review. After eligibility criteria were applied and duplicate records removed, a total of six randomized controlled clinical trials on the effect of polyunsaturated fatty acids (PUFAs) on clinical manifestations and inflammatory parameters in patients with Sjögren’s syndrome were included. Figure 1 shows the screening flow diagram in this review.

Of these six studies, five studies reported the effect on clinical manifestations, of which one study reported the effect of omega-3 supplementation, one study reported the effect of omega-6 supplementation, and three studies reported the effect of a combination of polyunsaturated fatty acids. Additionally, three studies reported the effect on inflammatory parameters, of which one study reported the effect of omega-3 supplementation, one study reported the effect of omega-6 supplementation, and one study trial reported the effect of flaxseed oil. A total of 290 participants were included in the studies analyzed, of whom 204 received PUFA supplementations, and 150 received placebos.

### 3.2. Effect of Polyunsaturated Fatty Acids (PUFAs) on Fatigue and Clinical Manifestations

Supplementation with polyunsaturated fatty acids, specifically gamma-linolenic acid (GLA), did not show a significant effect on fatigue [27]. In addition, several clinical trials showed a significant improvement in dry keratoconjunctivitis (dry eye): two clinical trials with Efamol supplementation, which consisted of a combination of polyunsaturated fatty acids—linolenic acid, gamma-linolenic acid, and saturated and monounsaturated fatty acids—with an intervention dose of 112 mg of LA and 15 mg of GLA (taken twice a day); and another clinical trial, which used an intervention dose of 3 g of Efamol (six capsules/day) and which used a combined omega-6 supplementation, which included linoleic acid and gamma-linolenic acid [19,20,21]. However, a randomized, double-blind, placebo-controlled clinical trial showed that supplementation with the intake of polyunsaturated fatty acids, with the intervention dose of 800 mg or 1600 mg for 6 months, had no significant effect on dryness [27]. Efamol supplementation also appears to have no significant effect on corneal sensitivity and nuclear chromatin in connective tissue epithelial cells [20].

Regarding the effect of PUFA supplementation on xerostomia (dry mouth), results from clinical trials evaluating this clinical manifestation showed no statistically significant change. One trial was biased by the fact that it was randomized in a ratio of 2:1, favoring a greater number of participants in the intervention group (with the treatment based on omega-3) compared with the placebo group. Additionally, a dietary intervention based on PUFA supplementation, with low intervention doses (3 g of Efamol; 1 g of flaxseed, 450 mg of DHA, 163 mg of vit. E, 20 mg of tocopherol; 800 mg of 1600 mg of GLA) for 3 to 6 months did not show significant change in fatigue or other clinical manifestations [26,27]. Regarding stimulated salivary flow or unstimulated salivary flow, one clinical trial showed no significant differences between the intervention group and the placebo groups [26].

### 3.3. Effect of Polyunsaturated Fatty Acids (PUFAs) on Inflammatory Parameters

With regard to inflammatory parameters, a clinical trial analyzing the effect of omega-6 supplementation on prostaglandin E1 (PGE1) showed a significant increase in PGE1 levels, after 1 month of intervention, with a dose of 112 mg of LA and 15 mg of GLA. However, a decrease in PGE1 levels was observed at the end of the intervention [19]. In another study, the supplementation with Efamol showed no significant changes in tear lysozyme levels [20]. In ocular inflammation, there was a significant reduction in ocular surface inflammation before and after treatment with flaxseed oil supplementation (rich in omega-3), the intervention doses of which were 1 g of flaxseed oil, 950 mg of synthetic mineral oil, 50 mg of GLA (one capsule per day), 950 mg of synthetic mineral oil, and 50 mg of GLA (two capsules per day) [23]. The administration of 3 g Efamol also showed a significant increase in the binding of oleic acid (dihomo-gamma-linolenic acid) to erythrocyte phospholipids, comparing before and after the 8-week treatment [21]. Table 1 summarizes the results of randomized and controlled clinical trials of the effect of polyunsaturated fatty acid supplementation on clinical manifestations and inflammatory parameters in individuals with Sjögren’s syndrome.

## 4. Discussion

This narrative review provides an overview of the effect of polyunsaturated fatty acid (PUFA) supplementation on clinical manifestations and inflammatory parameters in patients with Sjögren’s syndrome. Also, this review discusses the possible mechanism of action of PUFAs in Sjögren’s syndrome, contributing as a tool for better clinical practice and thus improving the quality of life of Sjögren’s syndrome patients.

According to Hong K. et al., omega-3 fatty acids may play an important role in rheumatic diseases such as rheumatoid arthritis (RA) and systemic lupus erythematosus. (SLE). According to this review, using a Mendelian randomization analysis, it is suggested that omega-3 fatty acids can be an effective complementary therapy in reducing the risk and activity of these diseases, as well as in mitigating inflammatory biomarkers. Hong K. et al. suggest that elevated levels of omega-3 are associated with a lower risk of disease occurrence. The suppression of inflammation by omega-3 can be explained by the reduction in the production of inflammatory cytokines such as cytokine-1 (IL-1) and Tumor Necrosis Factor alpha (TNF-α) and the increased activation of the pro-inflammatory transcription factor of activated B cells (NFKB) through peroxisome proliferator-activated receptor gamma (PPAR-γ). Additionally, they state that patients with RA who regularly take fish oil supplements are more likely to achieve disease remission and use fewer non-steroidal anti-inflammatory drugs (AINES). In individuals with SLE, it is noted that omega-3 fatty acids tend to reduce the risk of the disease by modulating inflammatory mediators and C-reactive protein (PCR) levels, leading to better disease control. Furthermore, it has been demonstrated that omega-3 supplementation tends to improve endothelial function and reduce oxidative stress in patients with SLE [30].

Patients with Sjögren’s syndrome report changes in quality of life, particularly in fatigue and sleep disturbance [27]. In addition, this disease causes changes in the epithelial cells of the ocular conjunctiva [20], leading to dry keratoconjunctivitis [19,20,21] and xerostomy [26,27]. Sjögren’s syndrome negatively affects inflammatory parameters, leading to alterations in inflammatory biomarkers, such as C-reactive protein (PCR), cytokine-6 (IL-6), acute phase proteins, and soluble intercellular adhesion molecule 1 [25].

Several nutritional therapy approaches have been studied to understand the effect of food or nutritional supplement intake on inflammatory parameters and clinical manifestations in Sjögren’s syndrome patients [19,20,21,22,23,23,24,25,26,27]. However, studies published in the literature have shown controversial results. In this context, our literature review aims to evaluate the evidence on the effects of PUFA, specifically omega-3 and omega-6, on inflammatory parameters, fatigue, and clinical manifestations in patients with Sjögren’s syndrome. The results of our study review showed a limited number of clinical trials on this topic. In this sense, this is a theme that should be investigated by the scientific community to better understand the impact of polyunsaturated fatty acid supplementation on Sjögren’s syndrome.

The current evidence shows that PUFA supplementation can improve dry keratoconjunctivitis (dry eye) and inflammatory parameters, such as erythrocyte levels, total plasma phospholipid levels, and ocular surface inflammation. We found no significant changes in other inflammatory parameters and clinical manifestations, such as the tear prostaglandin E1 content, tear lysozyme concentration and salivary secretion rate, stimulated and unstimulated salivary flow, fatigue, and xerostomy. Two trials using Efamol supplementation based on a combination of oils containing fatty acids (linoleic acid, gamma-linolenic acids; saturated and monounsaturated fatty acids) showed promise in improving symptoms such as nausea, reduced stool consistency, temporary flushing of the face and neck, the slight sensation of heat, and an increased heart rate during the intervention. The conflicting results found in this work may be due to the differences in study design, samples, supplementation, doses, intervention time, pharmacological therapy, and other factors.

PUFAs have been shown to possess a beneficial effect on inflammation, lipid mediator synthesis, immune regulation, and oxidative stress [25,29,31]. According to studies, the possible mechanism of action responsible for these biological effects might be due to the anti-inflammatory properties of eicosapentaenoic acid (EPA) and docosahexaenoic acid (DHA). The administration of high concentrations of EPA and DHA in cell membranes can lead to a decrease in the production of eicosanoids derived from arachidonic acid (AA) [25,28,32,33,34]. The decrease in AA in cell membranes may be due to the competition between PUFA (EPA and DHA) and AA for incorporation into cell membrane phospholipids, which can lead to the inhibition of the cycloxigenase-2 (COX-2) and lipoxigenase-5 (5-LOX) enzymes and, on the other hand, to the competition with AA for the metabolism of enzymes cyclooxygenase (COX), lipoxygenase (LOX), or cytochrome P450 [28]. According to Djuricic et al., the anti-inflammatory properties of EPA and DHA also reduce the activation of the pro-inflammatory transcription factor of activated B cells (NFKB) in response to inflammatory stimuli [25]. In another study, the authors showed that the activation of the receptor coupled to plasma membrane protein G (GPR120) by DHA reduces NFKB activation in macrophages, thereby reducing the production of inflammatory cytokines and, consequently, decreasing the inflammatory process [35].

According to López-Vicario et al., and Serhan et al., EPA and DHA are lipid mediators that act as precursors for the synthesis of other compounds [36,37,38,39], such as resolvins and protectins [37]. These compounds modulate neutrophil infiltration, reduce the production of inflammatory cytokines and oxidative stress, reduce inflammation, and thus attempt to restore immune system homeostasis [25,31]. In addition, studies have shown that omega-3 fatty acids inhibit the activation of immune cells, macrophages, and innate and adaptive immune systems [31]. In the innate immune system, omega-3 fatty acids and their metabolites promote reduced cytokine production and increased phagocytic capacity [31]. Supplementation with PUFA appears to regulate the adaptive immune system by mediating T and B lymphocyte responses [31]. The T lymphocytes control the differentiation of native helper T cells (CD4+ T) into Type 1 T helper (TH1) and Type 7 T helper (TH7) effector cells, reducing cytokine-2 (IL-2), TNF-α, and Interferon-gamma (INF-γ) in cytotoxic T lymphocytes (CD8). Lymphocytes/B cells tend to potentiate the decrease in B cell activation, but due to the limited evidence and the controversial nature of this topic, further studies should be conducted [31]. Omega-3 supplementation can also reduce the activity of natural killer cells (NK), depending directly on the age of the individual, with the greatest effect being seen in people over 55 years of age [40,41].

Based on Djuricic et al.’s study, omega-3 and omega-6 fatty acids appear to play an important role in regulating the oxidative process. Lipid peroxidation occurs by three cellular mechanisms, namely, free radical mediation (OH, O_2_, H_2_O_2_, etc.), oxidation by enzymatic metabolism (COX and LOX enzymes), and the oxygenation of non-enzymatical metabolism not mediated by free radicals [25]. The overproduction of free radicals alters the redox potential of the cell, affecting cell structures, such as membranes, proteins, lipids, lipoproteins, and deoxyribonucleic acid (DNA) [25]. The potential mechanism of action by which PUFA regulates the oxidative process may involve the regulation of LOX and COX enzymes. Inflammation and oxidative stress are closely linked, with inflammation promoting oxidative stress, which, in turn, activates inflammatory stress pathways [25].

## 5. Conclusions

The findings of this literature review provide current evidence from six randomized clinical trials available in the scientific literature on PUFA supplementation in Sjögren syndrome. According to the results, PUFA supplementation has a potential effect on clinical manifestations, such as keratoconjunctivitis and inflammatory parameters including total plasma phospholipid levels, erythrocyte phospholipids of plasma fatty acids, and eye surface inflammation in patients with Sjögren syndrome. PUFA can also contribute as an anti-inflammatory and antioxidant agent. However, the results of this work should be validated through systematic reviews and meta-analyses to better understand the potential effect of PUFA in Sjögren’s syndrome. Additionally, the inclusion of observational studies could provide important information that can be applied in clinical practice. However, due to the lack of studies on the effect of polyunsaturated fatty acid supplementation in Sjögren syndrome, more research is needed to verify its efficacy. Furthermore, the dose–response relationship should also be investigated in order to better understand its potential role in disease treatment strategies.

This study has a limitation due to the small number of randomized controlled studies on the effect of PUFA in Sjögren’s syndrome patients, particularly regarding clinical manifestations and inflammation parameters. A substantial number of peer-reviewed articles were excluded, taking into account medical drug therapy used for syndrome treatment or a lack of clear information regarding supplementation. In this context, further randomized controlled trials and observational studies should be included in future research to explore the long-term effects of PUFA supplementation on clinical manifestations and inflammation parameters.

## Figures and Tables

**Table 1:** A summary of the effects of polyunsaturated fatty acid supplementation on clinical manifestations and inflammatory parameters in individuals with Sjögren’s syndrome.

References	Study Design	Sample	Intervention	Results
[25]	Prospective, randomized, and placebo-controlled and double-blind clinical trial	*N* = 61 individuals (4 men and 57 women) Average age of 61 years	Intervention group: TheraTears Nutrition^®^ (1 g of flaxseed oil, 450 mg of EPA, 300 mg of DHA, 163 mg of vit. E, and 20 mg of tocoferol). Total: 1.750 mg of *n* − 3 Placebo group: Capsules (2.064 mg wheat germ oil, containing 144 mg linolenic acid (*n* − 3) and 3.63 mg vitamin E) 3 months	There were no significant differences between groups in unstimulated salivary flow (*p* = 0.38), and in the stimulated salivary flow rate (*p* = 0.35) There were no significant differences between groups in the self-perception of symptoms of dry mouth (*p* = 0.82)
[23]	Double-masked and controlled randomized clinical trial	*N* = 40 individuals (37 women; 3 men) Average age of 36.6 years	Intervention group: Linoleic acid (LA) (112 mg) with gamma-linolenic acid (GLA) (15 mg) at 2 doses/day, in the tear content of PGE1 Placebo group: 2 doses/day, in the tear content of PGE1 1 month	There were no changes in the enzyme content (PGE1) of tears (*p* > 0.05) ↑ PGE1 (T1 vs. T0) (*p* < 0.01) ↓ PGE1 (T2 vs. T0) (*p* < 0.01) Significant improvement symptoms in burning; dryness; and feeling of a foreign body (T1 vs. T2) (*p* < 0.01)
[28]	Randomized, double-blind, placebo-controlled clinical trial	*N* = 87 individuals (79 women; 8 men) Average age of 62 years	Intervention group: GLA (extracted from evening primrose oil): 800 mg or 1600 mg/day, capsules Placebo group: Corn Oil Capsules 6 months	There were no significant changes in fatigue, dryness, or pain in the eyes and mouth (*p* > 0.05)
[24]	Randomized, double-blind, placebo-controlled clinical trial	*N* = 28 individuals (24 women; 4 men) Average age of 51 years	Intervention group: 3 g of Efamol (seed oil consisting of 73% cis-linolenic acid; 9% GLA; 18% saturated and monounsaturated fatty acids)/day (6 capsules) Placebo group: Capsules of identical appearance 8 weeks	Significant improvement in dry keratoconjunctivitis (before vs. after treatment) (*p* < 0.05) No significant changes in the lacrimal lysozyme concentration or secretion rate during treatment (*p* > 0.05) No significant changes in xerostomy (*p* > 0.05) ↑ erythrocyte fatty acid-binding phospholipids (20: 3n6-ac. Dihomo-gamma-linolenic acid (DGLA)) (*p* < 0.001) ↓ erythrocyte phospholipids of oleic acid (binding acids-18: 1n9) (before vs. after treatment) (*p* < 0.02)
[26]	Randomized, double-blind, placebo-controlled clinical trial	*N* = 36 individuals (33 women; 3 men) (34–76 years) Average age of 55 years	Intervention group: 3 capsules (2/day): 1 capsule of Efamol (500 mg, containing 73% cis-linoleic acid, 18% saturated and monounsaturated fatty acids, and 9% GLA); 1 capsule containing 13.6 international units of vit. E.; 1 capsule of Efavit (125 mg vit. C, 25 mg pyridoxine, 25 mg of niacin, 5 mg zinc sulphate) Placebo group: Capsules with identical appearance, 3 capsules twice/day 3 weeks	Significant improvement in dry keratoconjunctivitis (*p* = 0.03) There were no significant changes in corneal sensitivity, tear enzymes, and nuclear chromatin in connective tissue epithelial cells (*p* > 0.05)
[29]	Randomized, double-blind, placebo-controlled clinical trial	*N* = 38 individuals (women) (21–55 years) Average age of 38 years	2 intervention groups: Group 1: 1 capsule of 1 g linseed oil (OL) + 1 placebo capsule identical to OL (950 mg of synthetic mineral oil and 50 mg of primrose oil) (GLA) Group 2: 2 OL capsules Placebo group: Group 3: 2 placebo capsules 180 days	↓ significant inflammation of the eye surface (before vs. after treatment) (*p* < 0.05)

EPA, eicosapentaenoic acid; LA, linoleic acid; DHA, docosahexaenoic acid; DGLA, dihomo-gamma-linolenic acid; GLA, gamma-linolenic acid; Vit. C, vitamin C; Vit. E, vitamin E; PGE1, prostaglandin E1; OL, flax oil; T0, start of study; T1, after 1 month of treatment; T2, after 15 days of discontinuation of treatment; ↓, decrease; ↑, increase.

## Data Availability

Not applicable.
